# Association Analysis of HIMP and SHIMP Quantitative Parameters in Patients With Vestibular Neuritis and Healthy Participants

**DOI:** 10.3389/fneur.2021.748990

**Published:** 2021-10-27

**Authors:** Feiyun Chen, Zichen Chen, Yuzhong Zhang, Xinyu Wei, Huandi Zhao, Juan Hu, Ying Cheng, Xiaoyong Ren, Qing Zhang

**Affiliations:** ^1^Department of Otorhinolaryngology Head and Neck Surgery, Second Affiliated Hospital of Xi'an Jiaotong University, Xi'an, China; ^2^Department of Otorhinolaryngology Head and Neck Surgery, Xinhua Hospital, Shanghai Jiaotong University School of Medicine, Shanghai, China; ^3^Shanghai Jiaotong University School of Medicine Ear Institute, Shanghai, China; ^4^Shanghai Key Laboratory of Translational Medicine on Ear and Nose Diseases, Shanghai, China

**Keywords:** HIMP, SHIMP, VOR, vestibular neuritis, association

## Abstract

**Background:** The Head Impulse Paradigm (HIMP) and Suppression Head Impulse Paradigm (SHIMP) are objective, quantitative methods that directly test the vestibulo-ocular reflex (VOR) and are increasingly becoming a standard in evaluating patients with vestibular disorders.

**Objective:** The main objective was to assess the correlations between HIMP and SHIMP parameters in patients with superior vestibular neuritis (VN) and healthy participants. Additionally, the correlations between the parameters of each method were analyzed.

**Methods:** A retrospective cohort, non-randomized study was designed. HIMP and SHIMP were performed on 40 patients with VN and 20 healthy participants (40 ears). HIMP and SHIMP parameters were measured and calculated. Pearson's or Spearson's correlations were used to establish the associations among them.

**Results:** A strong positive correlation was found between HIMP and SHIMP gain (Pearson's *r* = 0.957, *p* = 0.000), while strong negative correlations were detected between HIMP and SHIMP saccade amplitudes (*r* = −0.637, *p* = 0.000) and percentages of overt saccades (*r* = −0.631, *p* = 0.000). In HIMP, strong and moderate positive correlations were identified between gain and saccade amplitude (*R*^2^ = 0.726, *p* = 0.000) and gain and saccade percentage (*R*^2^ = 0.558, *p* = 0.000), respectively. By contrast, an extremely weak positive correlation was observed between gain and latency (*R*^2^ = 0.053, *p* = 0.040). In SHIMP, strong and moderate positive correlations were found between gain and saccade percentage (*R*^2^ = 0.723, *p* = 0.000) and gain and saccade amplitude (*R*^2^ = 0.525, *p* = 0.000), respectively, but no correlation was detected between gain and latency (*R*^2^ = 0.006, *p* = 0.490).

**Conclusions:** HIMP and SHIMP-related parameters were highly correlated (inter-method). Within each method (intra-method), moderate to strong correlations in VOR assessment were observed. These results further contribute to our understanding of the relationship between HIMP and SHIMP as well as to the diagnosis.

## Introduction

To facilitate clear vision, the vestibulo-ocular reflex (VOR) stabilizes the position of the eyes in a target space by generating delayed and equivalent eye movements that compensate for head rotations in the opposite direction; it is the basis of many routine vestibular tests (1). Among these, the video head impulse test (vHIT), a relatively recent clinical assessment tool, is used to assess the function of the semicircular canals, the angular acceleration detectors that initiate the VOR (2).

With the expansion of the clinical examination, the vHIT, having good sensitivity and specificity, is crucial in making or supporting the diagnoses of vestibular disorders, such as vestibular neuritis (VN), bilateral vestibular loss, and acoustic neuroma, as well as assessing vestibular compensation during the rehabilitation process (3).

Until recently, two testing protocols to evaluate the VOR using the vHIT were available: HIMP (Head Impulse Paradigm, or conventional vHIT) and SHIMP (Suppression Head Impulse Paradigm). They can be used complementarily to test semicircular canal function and to determine the level of vestibular function (2). The basic physiology underlying them is identical, and both paradigms can provide two parameters: VOR gain and corrective or catch-up saccades, although the main parameter for the HIMP was VOR gain before the SHIMP became available.

Accumulating evidence suggests that VOR gains are similar in both paradigms; nevertheless, the corrective saccades are expected to be complementary. In contrast to the HIMP, where corrective overt saccades indicate vestibular loss, and corrective covert saccades suggest vestibular compensation; in SHIMP, corrective or anti-compensatory saccades imply residual vestibular function (4, 5). Unfortunately, while these complementary findings are straightforward, they are difficult to interpret and validate from qualitative descriptions due to the lack of quantitative data. To date, few studies have comprehensively evaluated the parametric characteristics of VOR gain and saccades using inter- and intra-methods. To help address these gaps, our present study aimed, in patients with superior vestibular neuritis (SVN) and in healthy participants, to further elucidate the correlations between HIMP and SHIMP parameters.

## Materials and Methods

### Patient Selection

This retrospective study was approved by the institutional review board, and informed consent was signed by all study participants. A total of 40 patients with SVN and 20 healthy control participants were consecutively enrolled from May 2018 to August 2019.

The patient inclusion criteria were: meet the diagnostic criteria for VN (6); within 7 days of onset with continuous vertigo, nausea, vomiting, and spontaneous horizontal nystagmus on the healthy side; be diagnosed using video electronystagmography and v-HIT; and other central or peripheral vertigo was excluded via transcranial magnetic resonance imaging (MRI). All patients had normal gain of posterior semicircular canal to rule out injury of the inferior vestibular branch. Patients within 7 days of onset were selected because of the short onset time of 7 days, incomplete vestibular compensation, and obvious spontaneous nystagmus, which can provide a more definite diagnosis.

Patients that met any of the following criteria were excluded: central lesions detected via MRI; and have a history of other otologic diseases with hearing loss, vertigo, or middle ear pathology on clinical examination.

Healthy participants were included if: they had no history of deafness, tinnitus, or vertigo; their otoscopy, pure tone audiometry, and acoustic immittance tests were normal; and were willing to participate in the study.

The exclusion criteria were the presence of: external, middle, or inner ear diseases; dizziness, vertigo, or headache; and cerebrovascular and visual system diseases (optic nerve, ophthalmoplegia, etc.).

### HIMP and SHIMP Protocols

HIMP and SHIMP were measured using video goggles (ICS Impulse; Otometrics, Denmark). More specifically, the patient was seated and fixed the video goggles, which were coupled with a speed sensor and laser, on their head. The patient was asked to always look at a target 120 cm away. The examiner first calibrated the equipment, after which she stood behind the patient and turned the patient's head with both hands to the left and right horizontally. The left and right horizontal semicircular canals were measured 20 times each using passive, sudden, and unpredictable head turns that were fast (150–200 ms) and had low amplitudes (15–20 degrees), medium angular velocities (150–250 degrees/sec), and high angular accelerations (3000–6000 degrees/sec^2^). The fixed target on the wall was then removed, and the patient was asked to fix his/her eyes on the laser point emitted by the goggles. The examiner turned the patient's head 20 times each to the left and to the right. It suggest that the difference in the VOR gain between outward and inward thrusts was slight, both methods are acceptable for clinical use (7). In this paper, HIMP and SHIMP impulse both start at the center to minimize the participants prediction. The 20 head turns on either side had to be effective.

### Observed Parameters and Calculation Methods

HIMP and SHIMP gains, latencies, amplitudes, and percentages of overt saccades were recorded in healthy participants and patients. If no corresponding anti-compensatory saccade, latency, or peak velocity was detected, the gain value was excluded from the average gain.

To quantify VOR gain, we calculated the areas under the curves from the onset of the head impulse to the back crossing of zero-velocity (8). We defined VOR gain as the ratio of the area under the eye velocity (degree of eye rotation) to the area under the head velocity (degree of head rotation). The average VOR gain for each side was calculated as the sum of the VOR gains for each trial divided by the number of trials. We automatically excluded the impact of covert saccades (occurred before zero-velocity was crossed or the end of the head movement) using the manufacturer's own methods (i.e., the de-saccades algorithm).

**Figure 1:**
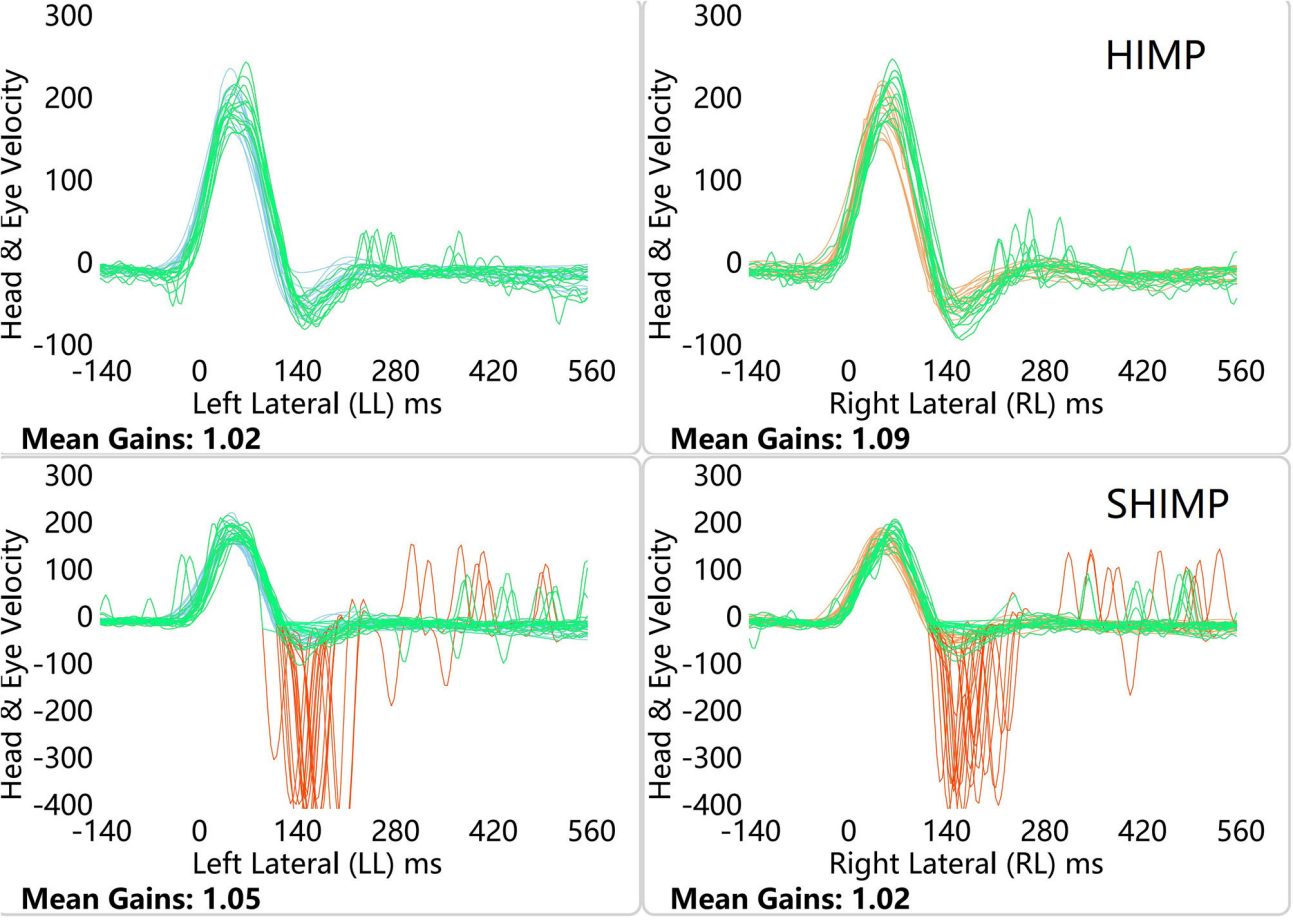
HIMP and SHIMP graphs in a healthy participants. The image above is bilateral HIMP and the image below is SHIMP of healthy participants. In healthy participants, a small number of weak saccades in the left and right sides were observed in the HIMP, while multiple anti-compensatory saccades were detected in the SHIMP.

**Figure 2:**
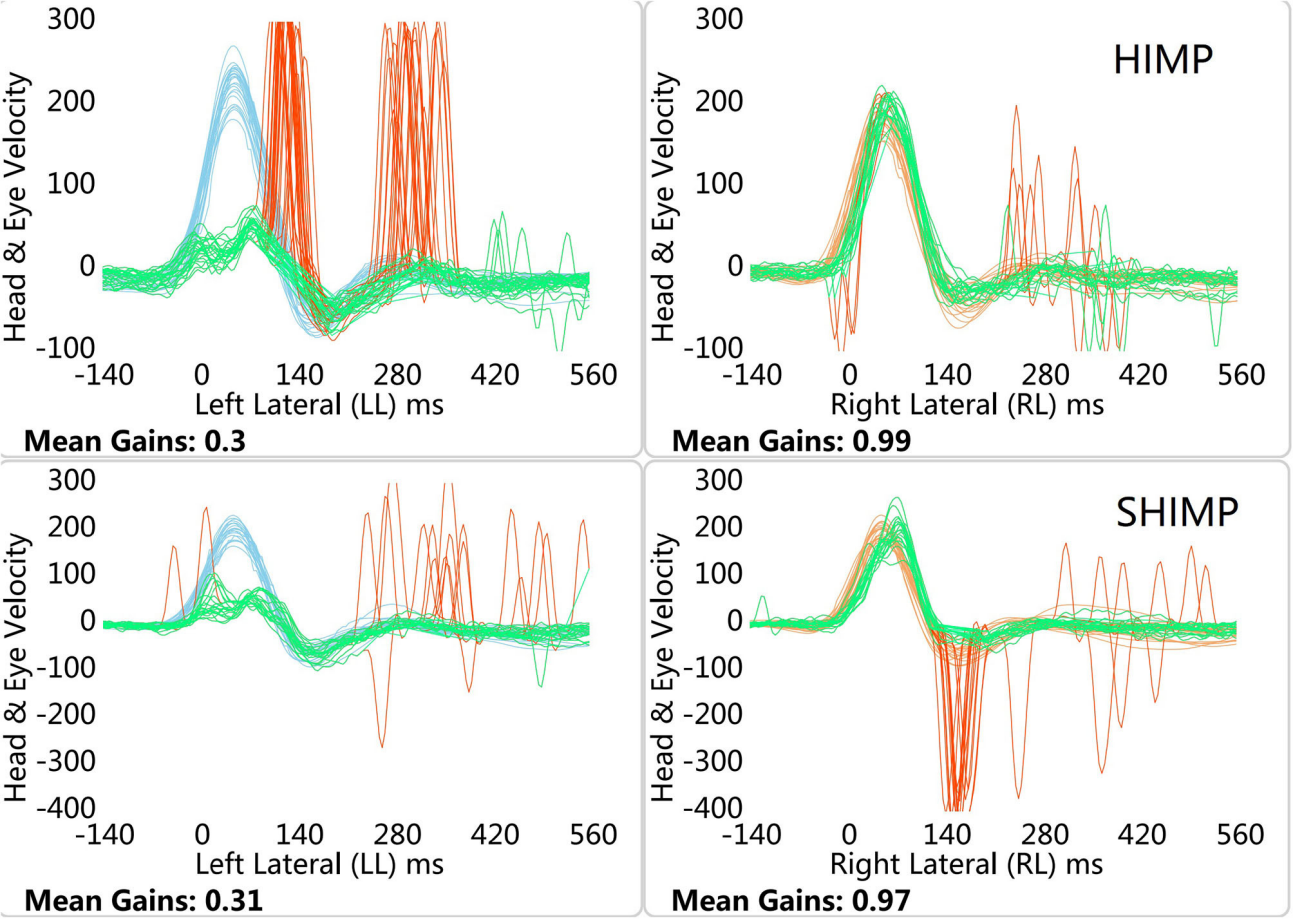
HIMP and SHIMP graphs in left VN patient. The image above is bilateral HIMP and the image below is SHIMP of VN patient. In patients with VN, no catch-up saccades were found on the healthy side in the HIMP, while they were detected on the affected side. In contrast to the HIMP, large anti-compensatory saccades were observed on the healthy side in the SHIMP, while none were found on the affected side.

Figures 1, 2 depict example data from the HIMP and SHIMP. VOR gains were calculated for SHIMP in the same way that they were calculated for HIMP. Peak saccade velocity was defined as the maximal velocity of the anti-compensatory saccade of SHIMP. Average peak saccade velocity was calculated as the sum of the saccade velocities divided by the number of trials. When no saccade was detected in a particular trial, the peak saccade velocity was considered as zero. We used average VOR gains, average peak saccade velocities, and average peak saccade velocity to peak head velocity ratios to assess patients' residual vestibular function, as described previously (5). Peak head velocity was defined as the maximal head velocity before zero was crossed again. Shen et al. (9) suggested that the peak saccade velocity to peak head velocity ratio is useful to eliminate false negatives caused by too slow head velocities.

### Statistical Analysis

Data are presented as means and ranges for continuous variables. The relationship between VOR gain values in HIMP and SHIMP were calculated using a paired sample *t*-test and linear regression. To evaluate the correlations between latency, amplitude, and saccade percentage between HIMP and SHIMP and within each protocol, Pearson's or Spearson's correlation coefficient and linear regressions were calculated. The statistical analyses were performed using SPSS v22.0 (IBM SPSS Inc., Armonk NY, USA). Two-tailed *p*-values < 0.05 were considered statistically significant.

## Results

### Patient Characteristics

The 40 enrolled patients included 16 males and 24 females aged 19–82 years with an average age of 46.3 ± 14.81 years. Twenty healthy age- and sex-matched adults (40 ears) were selected, which consisted of 7 males and 13 females aged 17–72 years with an average age of 42.2 ± 14.16 years. The age (*p* = 0.261) and gender (*p* = 0.623) distributions of the healthy participants and VN groups did not significantly differ (Table 1). The mean time since onset for each participant is 4.9 ± 1.7 days, and the intensity of spontaneous nystagmus is 7.7 ± 4.7 °/s.

**Table 1:** Demographic features of the healthy participants and vestibular neuritis (VN) groups.

	**Age (Years)**	**Sex**	
		**Male**	**Female**
Healthy participants	42.4 ± 14.6 (17–72)	7 (14 ears)	13 (26 ears)
VN	46.3 ± 14.8 (19–82)	16	24
*P*	0.261	0.623	

### Graph Characteristics

In healthy participants, a small number of weak saccades in the left and right sides were observed in the HIMP, while large anti-compensatory saccades were detected in the SHIMP (Figure 1). In patients with VN, no catch-up saccades were found on the healthy side in the HIMP, while they were detected on the affected side. In contrast to the HIMP, large anti-compensatory saccades were observed on the healthy side in the SHIMP, while none were found on the affected side (Figure 2).

### Relationship Between HIMP and SHIMP Parameters

**Figure 3:**
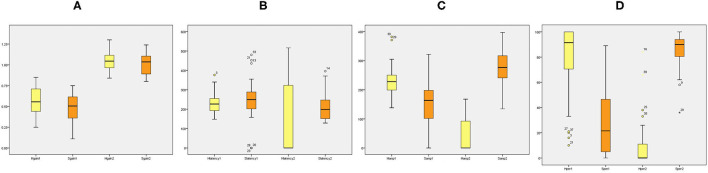
Box plot for comparison of parameters between VN patients and healthy participants. **(A)** HIMP and SHIMP gain of VN patients and healthy participants. **(B)** HIMP and SHIMP saccade latency of VN patients and healthy participants. **(C)** HIMP and SHIMP saccade amplitude of VN patients and healthy participants. **(D)** HIMP and SHIMP saccade percentage of VN patients and healthy participants. 1 = patients, 2 = healthy. Yellow indicates HIMP parameters and orange indicates SHIMP parameters.

**Figure 4:**
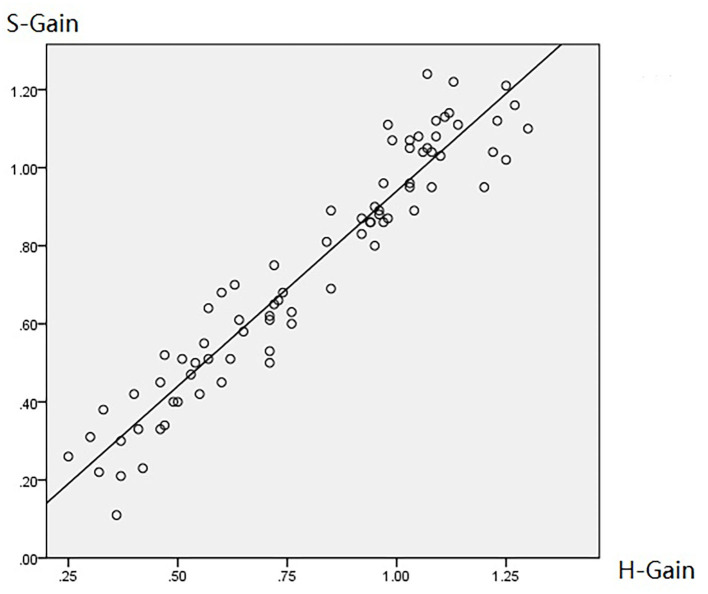
Diagram of HIMP and SHIMP gain correlation. HIMP and SHIMP gain were highly positively correlated (*r* = 0.957, *p* = 0.000; Table 3; this figure).

**Figure 5:**
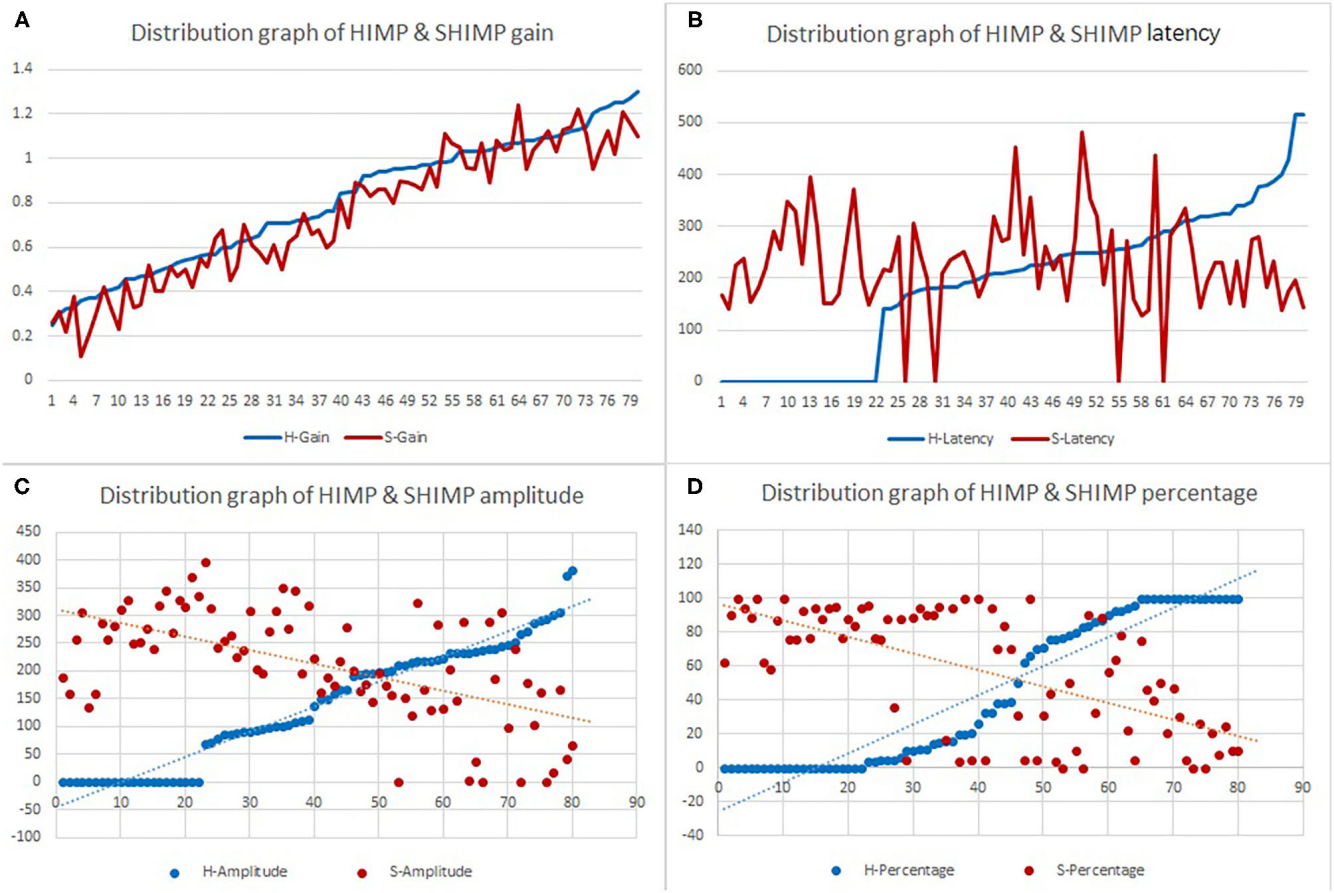
Scatter plot demonstrating the associations between HIMP and SHIMP parameters in all participants. **(A)** Gain between HIMP and SHIMP were highly positively correlated (*r* = 0.957, *p* = 0.000). **(B)** HIMP and SHIMP latencies were not correlated (*r* = 0.077, *p* = 0.499). **(C)** Saccade amplitudes between HIMP and SHIMP were strongly negatively correlated (*r* = −0.637, *p* = 0.000), as were the **(D)** percentages of overt saccades between HIMP and SHIMP (*r* = −0.631, *p* = 0.000).

Table 2 summarizes the associations between HIMP and SHIMP parameters in VN patients and healthy participants. As indicated above, in VN patients, HIMP gain, amplitude, and percentage of overt saccades were significantly larger than those of SHIMP on the affected side. In healthy participants, HIMP gains were significantly larger than those of SHIMP. The saccades amplitude, and percentage of overt saccades were significantly lower than those of SHIMP. Both HIMP and SHIMP, there were no statistical differences in saccades latency between patients and healthy participants. Figure 3 demonstrated the Box plot for comparison of parameters between VN patients and healthy participants. HIMP and SHIMP gains of patients were significantly lower than those of healthy participants. There is no difference of latency. SHIMP saccade amplitude and percentage were lower in patients than in healthy participants, whereas, HIMP saccade amplitude and percentage were higher in patients than in healthy participants. HIMP and SHIMP gain were highly positively correlated (*r* = 0.957, *p* = 0.000; Table 3; Figure 4). By contrast, HIMP and SHIMP amplitudes and percentages of overt saccades were both strongly negatively correlated (Table 3; Figure 5). Latencies did not differ (*t* = −0.498, *p* = 0.621; Table 2) or correlate (*r* = 0.077, *p* = 0.499; Table 3; Figure 5) between the two methods. It should be noted that, most of the healthy participants and patients were female. The head hand position would make the hair move, so that the goggles might slip slightly from the face or skull, which generate high gains (>1) (10). The perfect gain of the VOR is 1, but it's hard to achieve. In some patients, the high gain may be related to skin slipping and unskillful operation in early time.

**Table 2:** HIMP and SHIMP parameters of the affected side in VN patients and healthy participants.

	**Gain**	**Latency (ms)**	**Amplitude (°/s)**	**Percent (%)**
HIMP(P)	0.55 ± 0.15	233.68 ± 50.99	231.80 ± 52.92	78.48 ± 27.95
SHIMP(P)	0.48 ± 0.16	242.65 ± 108.43	150.60 ± 89.02	27.95 ± 25.73
*t*	5.389	−0.498	4.287	9.071
*p*	0.000	0.621	0.000	0.000
HIMP(N)	1.05 ± 0.11	149.85 ± 180.57	43.98 ± 51.23	9.33 ± 18.05
SHIMP(N)	1.01 ± 0.12	214.13 ± 71.27	273.83 ± 59.92	86.48 ± 13.41
*t*	3.424	−1.907	−18.787	−24.497
*p*	0.001	0.064	0.000	0.000

*This table summarizes the associations between HIMP and SHIMP parameters in VN patients and healthy participants. P = VN patients, N = healthy participants*.

**Table 3:** Correlation between HIMP and SHIMP parameters in all participants (mean ± SD; *n* = 80).

	**Gain**	**Latency (ms)**	**Amplitude (°/s)**	**Percent (%)**
HIMP	0.80 ± 0.29	191.76 ± 138.42	137.89 ± 107.75	43.90 ± 41.92
SHIMP	0.74 ± 0.30	228.39 ± 92.29	212.21 ± 97.61	57.21 ± 35.82
*r*	0.957	0.077	−0.637	−0.631
*p*	0.000	0.499	0.000	0.000

### Interrelations Between HIMP Parameters

Table 4 summarizes the associations between HIMP and SHIMP parameters in all participants. In HIMP, gain was positively correlated with latency, amplitude, and percentage. By contrast, in SHIMP, the gain was positively correlated with amplitude and percentage; however, it was not correlated with latency.

**Table 4:** Correlational analysis of gain with other parameters in HIMP and SHIMP (*n* = 80).

	**H-gain**			**S-gain**			***R* ^**2**^**	***p***
	**H-latency**	**H-amplitude**	**H-percent**	**S-latency**	**S-amplitude**	**S-percent**	**0.915**	**0.000**
*R* ^2^	0.053	0.726	0.558	0.006	0.525	0.723		
*p*	0.040	0.000	0.000	0.490	0.000	0.000		

**Figure 6:**
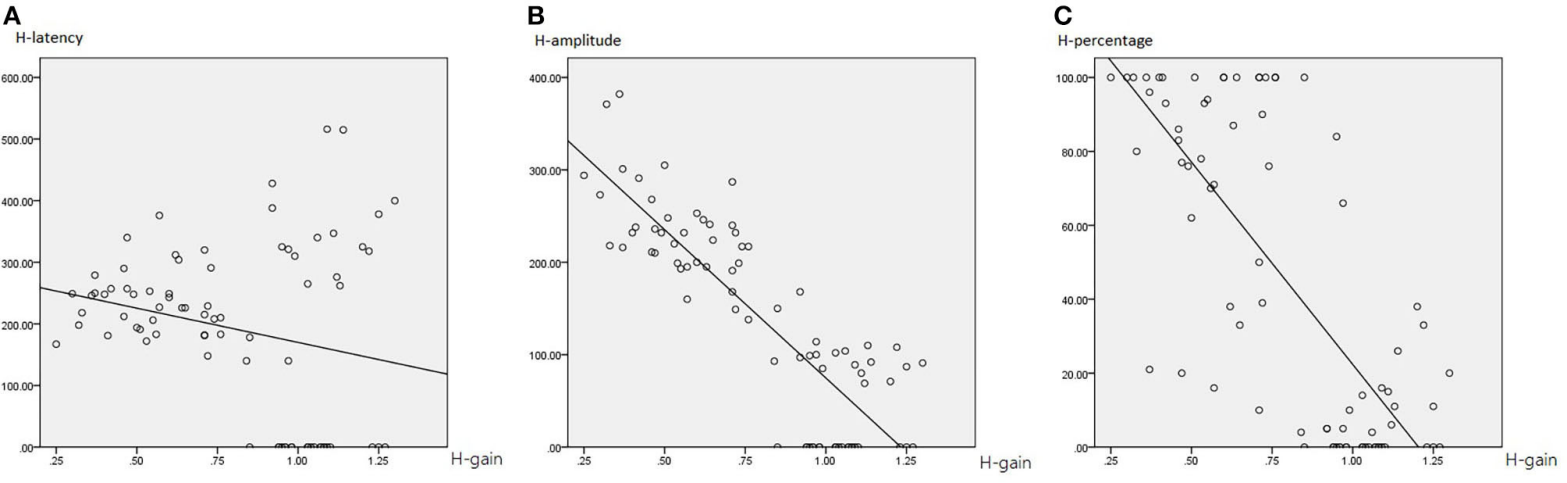
The correlation between HIMP gain and saccade latency **(A)**, amplitude **(B)**, and percentage **(C)**. The gain and latency were weakly negatively correlated. However, the saccade amplitude and percentage had strong and moderate negative correlations, respectively, with gain.

More specifically, in HIMP (Table 4; Figure 6), gain and latency were weakly negatively correlated (Spearson *R*^2^ = 0.053, *p* = 0.040). However, the saccade amplitude (*R*^2^ = 0.726, *p* = 0.000) and percentage (*R*^2^ = 0.558, *p* = 0.000) had strong and moderate negative correlations, respectively, with gain.

### Interrelations Between SHIMP Parameters

**Figure 7:**
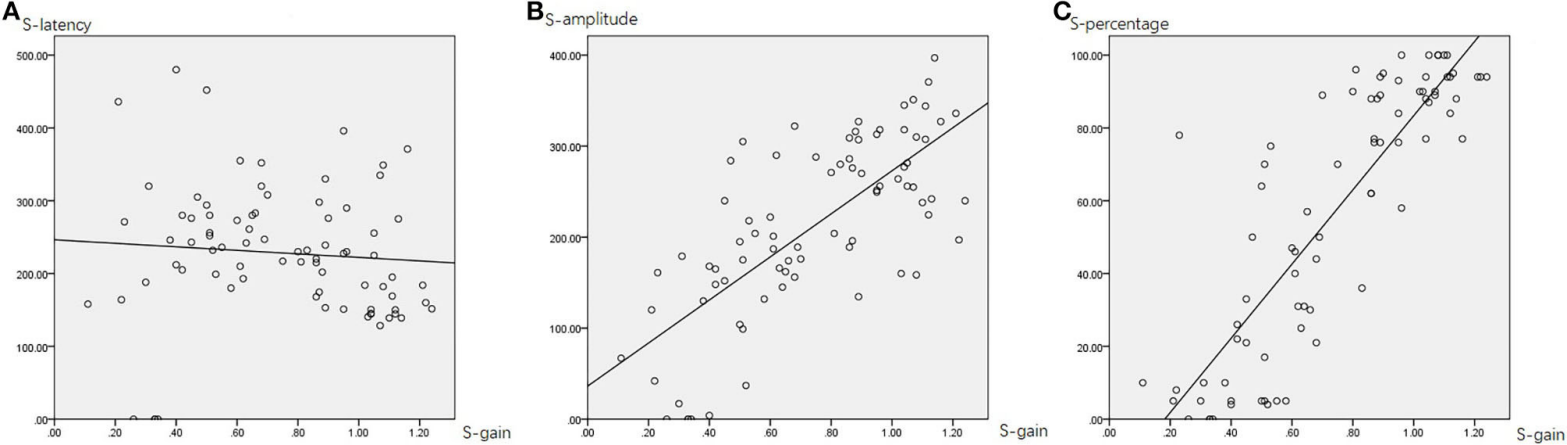
The correlation between SHIMP gain and saccade latency **(A)**, amplitude **(B)**, and percentage **(C)**. The gain and latency did not correlate. Nevertheless, saccade amplitude and percentage were moderately and strongly positively correlated, respectively, with gain.

Table 4 summarizes the associations between HIMP and SHIMP parameters in all participants. In SHIMP (Table 4; Figure 7), gain and latency did not correlate (Spearson *R*^2^ = 0.06, *p* = 0.490). Nevertheless, saccade amplitude (*R*^2^ = 0.525, *p* = 0.000) and percentage (*R*^2^ = 0.723, *p* = 0.000) were moderately and strongly positively correlated, respectively, with gain.

## Discussion

Our main finding was that gain and saccade patterns within and between the two vHIT methods were moderately to highly correlated and can potentially complement each other. Our results may increase the understanding of the relationship between HIMP and SHIMP. As in previous article, HIMP and SHIMP are important to evaluate in patients with vestibular neuritis (11).

In HIMP, VOR pathway is complete in healthy participants. When head turns to one side, the eyes move in the opposite direction to maintain visual fixation on the fixed target. In patients with vestibular loss, the VOR does not correct for the head movement, so that fixation is taken off the target, requiring a compensatory saccade to regain the target. Similarly, In SHIMP, participants are required to follow a target from a head mounted laser moving with the head. VOR keeps the gaze fixed opposite direction to head, when the target moves to same direction to head. At the end of impulse, the healthy participants has to make a large saccade toward head to regain the target. This is an anti-compensatory saccade. For patients with unilateral vestibular loss, absent VOR does not drive their eyes off the head-fixed target at all during the head impulse, so at the end of the impulse the eyes are still on target, no saccade required.

The SHIMP is new and complementary measure of HIMP. Both these paradigms measure the VOR pathway of the horizontal semicircular canal. SHIMP is easy to perform. It doesn't need to turn the trunk, which is turned out to be an easier task for patients with language difficulties and young children. With ongoing advances in technology, the two vHIT-based paradigms potentially provide a comprehensive assessment of vestibular loss and function by measuring VOR gain and catch-up saccades in a “one-stop-shop” approach. However, the pathophysiological mechanisms and clinical impact underlying the saccadic response has yet to be elucidated.

### Correlation in VOR Gain Between HIMP and SHIMP

Multiple groups that mainly focus on VOR gain have confirmed that both HIMP and SHIMP yield the same diagnostic information regarding peripheral vestibular loss as well as the affected side. VOR gains have been demonstrated to be closely correlated with slightly lower SHIMP gains than HIMP gains in healthy participants (12, 13), dancers (14), and patients with unilateral or bilateral vestibulopathy (15). To the best of our knowledge, only one previous study (16) has compared the HIMP and SHIMP in acute vestibular neuritis (AVN); they reported that the gain between them were correlated as mentioned above. Thus, our findings are consistent with those of previous studies.

Although SHIMP is called suppression head impulse, it is not VOR suppression. Crane and Demer (17) found that the latency of VOR suppression with a visual target during high acceleration rotations was about 80 to 90 ms. During that latent time, the VOR is fully operational and uncontaminated by anything else. VOR suppression starts at the end of the head impulse. During the impulse for both HIMP and SHIMP it is just the semicircular canal drive to the eye muscles. Both HIMP and SHIMP give such excellent measures of VOR gain, so they are so similar and closely matched. SHIMP and HIMP gains are known to be closely correlated in both healthy participants and people with peripheral vestibular dysfunction (9, 16, 18). These studies have also reported consistently SHIMP gain reductions compared to HIMP gains. SHIMP gain reduction compared to HIMP gain has been postulated to refect cerebellar VOR suppression during SHIMP (14, 18).

In addition to the effect of technology, a recent study (16) in AVN patients found no correlation between the magnitude of spontaneous nystagmus and VOR gain. Rey-Martinez et al. (13) also reported that a gain difference exists in healthy participants without covert saccade or spontaneous nystagmus. Thus, from the perspective of the disease itself, VOR inhibition or other mechanisms may cause the differences in gain; however, this hypothesis remains unverified.

### Correlation in VOR Saccades Between HIMP and SHIMP

To the best of our knowledge, this study is the first to examine the quantitative relationship in saccade patterns between the two head impulse protocols. We found a strong negative correlation between HIMP and SHIMP not only in saccade amplitude (i.e., average peak saccade velocity) but also in the percentage of overt saccades. Accordingly, a trade-off seems to exist between vestibular loss and residual vestibular function.

A recent retrospective study (19) revealed that SHIMP saccade reappearance provides useful information regarding the value of the vestibulo-saccadic interaction as a new recovery strategy in patients with VN. Our quantitative results match the qualitative description provided by MacDougall et al. (5) and serve as a complement to their qualitative results to form an in-depth explanation.

To date, saccades can be elicited by SHIMP and HIMP, but they represent different opposing aspects of VOR function. SHIMP saccades are distinguishable from spontaneous nystagmus and HIMP saccades in the opposite direction and are therefore easily recognized without the complex off-line post-processing used to calculate PR scores (20).

Our findings add to the knowledge regarding the correlations between parameters and further suggest that the combined use of the two protocols may be helpful in comprehensive evaluations of vestibular function.

### Correlation Within HIMP and SHIMP Parameters

The HIMP is based on the assumption that, if the VOR system is intact, the patient will successfully maintain visual fixation on a target when the head is moved. The gain should be in a normal range close to 1.0 without pathological saccades. When VOR fails, the eye movements are unable to match the head movements; consequently, the patient will generate a catch-up saccade (i.e., covert and overt saccades) in the opposite direction of the moving head to recapture the target.

In clinical practice, accumulating evidence suggests that both VOR gain reduction and the presence of corrective saccades in HIMP indicates vestibular hypofunction. The most common response pattern is low gain concurrent with catch-up saccades; this typical counter-balance in patients with various pathologies is confirmed in our data. In SHIMP, the healthy participants do not suppress their VOR during the early stage (first 80 ms) of the head turn. Their eyes are driven by VOR off the fixed target. At the end of the impulse, the healthy participants has to make a large saccade (anti-compensatory saccade) to regain the target by correcting an eye position error. Accordingly, the gain should be in a normal range close to 1.0 with distinct anti-compensatory saccades. By contrast, patients with unilateral vestibular loss have low VOR gain and make small saccades or no saccades at all.

Two research groups have reported a mild correlation between SHIMP peak saccade velocity and HIMP gain. They state that the peak velocity of SHIMP saccades is a good alternative parameter to evaluate vestibular function in patients with AVN (16) and vestibular schwannoma (9). Our study is the first to identify moderate to strong positive correlations in intra-SHIMP gain and saccades. Our results provide additional evidence suggesting that these SHIMP parameters may be potential biomarkers in evaluating vestibular function.

## Complementary Correlations Between Intra-HIMP and Intra-SHIMP Parameters

Despite differences in their temporal frequencies, all vestibular function tests should be incorporated into a comprehensive vestibular function battery rather than used alone or as a substitute for other tests. These tests can be used to expand our understanding of vestibular disorders. Based on previous papers, the complementary relationships in vHIT fall into three levels: intra-method, inter-technique, and inter-method.

First, some have suggested that both low gain and catch-up saccades must be demonstrated to identify a peripheral vestibular disorder when evaluating the semicircular canals using vHIT. In clinical practice, however, beyond the typical VOR response pattern observed in patients with various pathologies, three other atypical patterns exist: normal gain with catch-up saccades, low gain without catch-up saccades, and high gain with normal catch-up saccades (21, 22). Fortunately, vHIT provides two complementary parameters (i.e., intra-method: gain and saccades) and thus might have clinical utility in certain scenarios (23–25).

Secondly, caloric-vHIT dissociation (inter-technique), where the caloric test is frequently more abnormal than vHIT, is a similar pattern that is now recognized to be a common pattern in Meniere's disease (26, 27).

Lastly, Gains are universally acknowledged to be sensitive indicators of vestibular loss. Saccades information may complement that VOR gain. By contrast, the main strength of SHIMP saccades as a complementary approach to HIMP is that they allow for the determination of residual vestibular function (4). As such, HIMP saccades and SHIMP methods are another novel complementary pattern (inter-method) to assess vestibular [lost and compensation or rehabilitation (16, 19)] function. vHIT continues to grow in clinical use and is a valuable complementary test within the peripheral vestibular test battery. Yet, further research is needed to determine its utility in various clinical scenarios.

## Limitations

The present study has several limitations. First, selection bias was unavoidable due to the retrospective study design, and it was a single institution study. Second, although we found evidence of relationships between the parameters, these relationships need further confirmation with larger sample sizes. Third, this paper focused on the relationships between HIMP and SHIMP parameters identified from a model of SVN, which has its specific location and pathophysiological mechanisms. There was no follow-up. Consequently, the results do not reflect the quantitative relationships between parameters during rehabilitation or chronic periods. Similarly, the findings cannot be extrapolated to all other diseases. Further studies are therefore necessary to extend our results. Finally, because of the small sample sizes and the focus of the study, we did not compare the differences between healthy controls and patients. Future studies on this matter are necessary to investigate.

## Conclusion

In this study, strong correlations between HIMP- and SHIMP-related parameters (inter-method) were detected, as were moderate to strong correlations within each method (intra-method) of VOR assessment. These results may increase our understanding of the relationship between HIMP and SHIMP as well as to the diagnosis.

## Data Availability Statement

The raw data supporting the conclusions of this article will be made available by the authors, without undue reservation.

## Ethics Statement

The studies involving human participants were reviewed and approved by Institutional Ethics Review Board of the Second Affiliated Hospital of Xi'an Jiaotong University. The patients/participants provided their written informed consent to participate in this study.

## Author Contributions

FC and ZC: study conception and design. FC, YC, JH, HZ, YZ, and XW: acquisition of data. FC: analysis and interpretation of data and drafting of manuscript. QZ and XR: critical revision. All authors read and approved the final manuscript.

## Funding

This study was supported by grants from the National Natural Science Foundation of China (Nos. 81970891, 82171137, and 81700915), the Shaanxi Major International Cooperative Project of China (No. 2020KWZ-019), and the Key R&D Projects in Shaanxi Province, China (No. 2018SF-189).

## Conflict of Interest

The authors declare that the research was conducted in the absence of any commercial or financial relationships that could be construed as a potential conflict of interest.

## Publisher's Note

All claims expressed in this article are solely those of the authors and do not necessarily represent those of their affiliated organizations, or those of the publisher, the editors and the reviewers. Any product that may be evaluated in this article, or claim that may be made by its manufacturer, is not guaranteed or endorsed by the publisher.
